# An outbreak of tuberculosis due to *Mycobacterium bovis* infection in a pack of English Foxhounds (2016–2017)

**DOI:** 10.1111/tbed.12969

**Published:** 2018-07-30

**Authors:** Conor O'Halloran, Jayne C. Hope, Melanie Dobromylskyj, Paul Burr, Kieran McDonald, Shelley Rhodes, Tony Roberts, Richard Dampney, Ricardo De la Rua‐Domenech, Nicholas Robinson, Danielle A. Gunn‐Moore

**Affiliations:** ^1^ Roslin Institute and Royal (Dick) School of Veterinary Studies University of Edinburgh Easter Bush, Midlothian UK; ^2^ Finn Pathologists Diss Norfolk UK; ^3^ Biobest Laboratories Edinburgh UK; ^4^ Animal and Plant Health Agency Addlestone, Surrey UK

**Keywords:** canine, *Mycobacterium bovis*, outbreak, tuberculosis

## Abstract

*Mycobacterium bovis* can cause tuberculosis (TB) in social mammals including lions, cattle and man, but canine infections are considered rare. In 2016/17 we investigated a *M. bovis* TB outbreak in a pack of approximately 180 Foxhounds within the bovine TB Edge Area of England. We employed a combination of immunological tests including an interferon gamma release assay (IGRA) and a serological assay (DPP VetTB, Chembio). Test‐positive hounds were euthanased and subjected to *post‐mortem* examination (PME). Overall 164 hounds were tested; 97 (59%) responded positively to at least one test. Eighty‐five (52%) dogs responded to *M. bovis* antigens by IGRA while only 21 (12.9%) had detectable serological responses. At PME three hounds (3.1%) had visible lesions (VL) due to *M. bovis* infection, later confirmed by culture. Samples from 24 non‐VL hounds were cultured and *M. bovis* infection was confirmed in a further three hounds (11%). This study is the first investigation and report of an outbreak of *M. bovis* TB in a canine species. We establish that, in principle, diagnostic tests used for identifying infected individuals of other species can effectively be used in the dog. Further work is urgently needed to establish the sensitivity and specificity of the testing approach used in this study for future clinical application.

## 1 INTRODUCTION

*Mycobacterium (M.) bovis* is one of the nine member species of the *Mycobacterium tuberculosis* complex (MTBC) which are capable of causing tuberculosis (TB), across a broad taxonomy of social mammal including but not limited to humans, lions, elephant and meerkats (Angkawanish et al., 2013; Brosch et al., 2002; Drewe, 2010; Miller et al., 2012; Parsons, Drewe, Gey van Pittius, Warren, & van Helden, 2013). Within this group of pathogens, *M. bovis* stands out as the least host restricted and thus has significant zoonotic potential (Broughan et al., 2013; Michel et al., 2013; Miller & Olea‐Popelka, 2013; Palmer, 2007). Disease in humans due to infection with *M. bovis,* termed “zoonotic TB” by the World Health Organisation (WHO), is a major global public health priority which resulted in nearly 150,000 cases and at least 12,500 deaths in 2010 worldwide (Ciszewski, Czekaj, Chojnacki, & Szewczyk, 2015; Dürr et al., 2013; Olea‐Popelka et al., 2017). In addition, the lack of diagnostic discrimination between *M. tuberculosis* and *M. bovis* in most human mycobacterial reference laboratories (MRLs) means that the true mortality and morbidity caused by zoonotic TB is possibly underestimated (Dürr et al., 2013; Olea‐Popelka et al., 2017).

Discriminating between TB caused by different species of the MTBC is challenging as all member species share identical sequences across the 16s rRNA gene and 99.5% sequence homology across the remainder of the genome (Brosch et al., 2002). The most discriminating features between the species at the nucleotide level are genomic deletions, termed regions of difference (RD; Teo, Cheng, Jureen, & Lin, 2013). These have been shown to encode a variety of different virulence factors, for example, RD‐1 is present on all MTBC mycobacteria other than *M. bovis* Bacillus‐Calmette‐Guérin (BCG) and *M. microti* and encodes for the immunodominant proteins and key virulence factors; early secreted antigenic target‐6 KDa (ESAT‐6) and culture filtrate protein‐10 KDa (CFP‐10) (Gao et al., 2004; Guinn et al., 2004; Junqueira‐Kipnis et al., 2006).

The human burden of disease is highest across Africa, South‐East Asia and the Western Pacific. In South Africa, dogs have been shown to become infected with *M. tuberculosis,* as diagnosed using human interferon gamma release assays (IGRA), when exposed to high risk humans, that is, those with active TB disease (Parsons, Warren, Ottenhoff, Gey van Pittius, & van Helden, 2012). Similarly, African wild dogs (*Lycaon pictus*) have been found to be infected with *M. bovis,* presumptively from hunting exposure (Ayele, Neill, Zinsstag, Weiss, & Pavlik, 2004). In the UK, however, the significance of *M. bovis* in companion animals is largely limited to domestic cats, were frequent diagnoses are made (Broughan et al., 2013; Gunn‐Moore, 2014; Pesciaroli et al., 2014; Rocha et al., 2017). Canine incidence of TB in the UK is currently considered to be rare, and almost all reported cases are limited to individual sporadic infection or small numbers of epidemiologically unrelated cases (Ellis et al., 2006; Gay et al., 2000; Liu, Weitzman, & Johnson, 1980; Park, Lim, Kwon, Bae, & Park, 2016; Parsons et al., 2012; Pesciaroli et al., 2014; Posthaus et al., 2011; Shrikrishna, de la Rua‐Domenech, Smith, Colloff, & Coutts, 2009; Snider, 1971; Szaluś‐Jordanow et al., 2016; Van Der Burgt, Crawshaw, Foster, Denny, & Schock, 2009).

Between the end of 2016 and July 2017, a large outbreak of *M. bovis* infection occurred in a kennel of working Foxhounds within the Edge Area of England; which is a buffer zone of intermediate bovine TB incidence separating the High and Low bovine TB Risk Areas and subject to additional surveillance and controls for this disease. This report describes the clinical features of the outbreak, the testing approach taken and the epidemiological aspects of the spread of disease as they are currently understood. Work to evaluate the testing approach further is currently ongoing and will be published separately. We go on to explain the new statutory controls which have been implemented in England as a result of this incident to minimize the low risk of spreading TB through the feeding of fallen stock to hounds in registered kennels.

## 2 OUTBREAK INVESTIGATION

### 2.1 Index case

The hunt kennel where this outbreak occurred is situated in the south of England, UK, and housed up to 180 working Foxhounds ranging in age from juvenile puppies (<10 weeks old, *n* = 32), young adults (up to and including a year old, *n* = 21) and adults up to 8 years old (median age of 4 years).

The kennel is situated within the designated Edge Area of bovine TB incidence. The hounds work across six counties, four of which are also within the Edge Area, and two in the Low Risk Area, up to three times weekly during the peak of their hunting season (August–April).

In a similar fashion to many hunt kennels, the hounds were predominately fed raw meat, permitted offal and bone from fallen stock (so called “flesh feeding”), as permitted under Animal By‐Products legislation (Article 18, Commission Regulation (EC) No. 1069/2009).

The index case (Case 0) occurred in December 2016. The pack had experienced an outbreak of upper respiratory tract disease in the previous weeks, resulting in a number of individual hounds suffering from reduced body condition, lethargy and poor appetite. Two of these hounds developed marked polyuria and polydipsia and died. Clinical suspicion at this time was of leptospirosis infection as routine vaccination was, and remains, unused and clinical signs were compatible (Rissi & Brown, 2014). The hounds were not subjected to any confirmatory diagnostic testing at this time and carcasses were disposed of. When a third hound, a 4 year old entire male with no previous history of ill health, developed anorexia, plus marked polyuria and polydipsia, blood samples were taken for routine haematology, serum biochemistry and leptospirosis IgG titre testing.

These tests revealed; an inflammatory leucocytosis (21.8 × 10^9^/L [reference interval, RI, 6–15 × 10^9^/L]) consisting of a mature neutrophilia (17 × 10^9^/L [RI 3.6–12 × 10^9^/L]), lymphopenia (0.4 × 10^9^/L [RI 0.7–4.8 × 10^9^/L]), monocytosis (3 × 10^9^/L [RI 0–1.5 × 10^9^/L]) and eosinopenia (0 × 10^9^/L [RI 2–10 × 10^9^/L]). Serum biochemical analysis showed a marked azotaemia (urea 18.7 mmol/L [RI, 1.7–7.4 mmol/L], creatinine 457 μmol/L [RI 40–132 μmol/L]), hypoalbuminaemia (18 g/L [RI 26–35 g/L]) and hypocalcaemia (total calcium 2.04 mmol/L [RI 2.30–3.00 mmol/L]). Serological testing returned only a very low positive combined leptospirosis IgG titre (1+), indicating that active leptospirosis was unlikely to be the cause of the azotaemia and other clinical signs.

**Figure 1:**
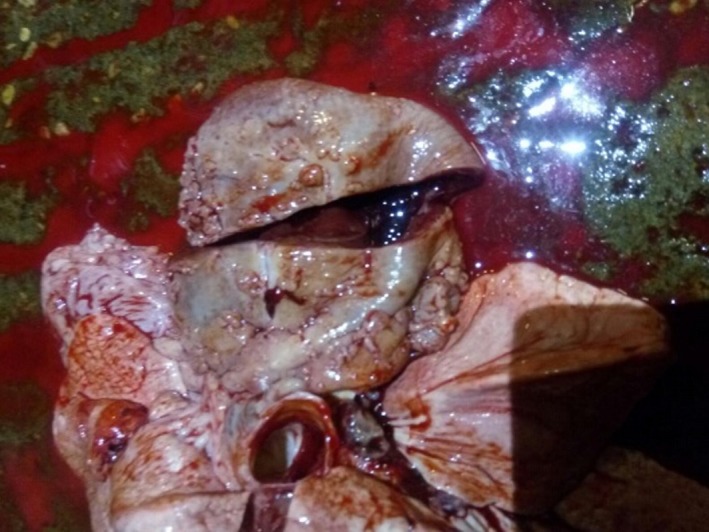
The left kidney of Case 0, removed at *post‐mortem* examination, the visceral and cut surfaces show diffuse discolouration and there are observable granulomas on the visceral surface

The dog was euthanased and subjected to *post‐mortem* examination (PME). Both kidneys were found to be grossly diffusely grey in colour and firmly textured. Across both the visceral and cut surfaces were multifocal to coalescing nodular, approximately round, areas containing hard white and soft black material (Figure 1). Representative samples were collected and fixed in formalin for histological evaluation by one of the authors (MD, FRCPath and experienced veterinary pathologist). Additional samples were frozen and later submitted for mycobacterial culture and PCR.

**Figure 2:**
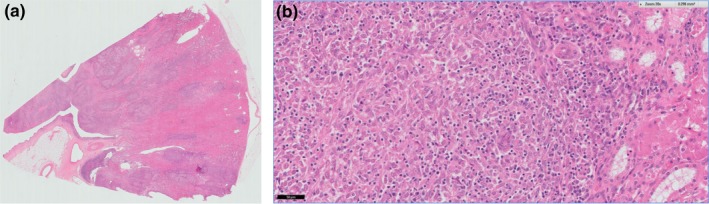
A section of kidney from Case 0 (shown grossly in Figure 1), stained with haematoxylin and eosin. Image A shows the section at low power (×10 magnification) where multifocal areas of pathology (granulomas) are visible. Image B is a high power view of the same section (×40 magnification) and shows numerous epithelioid macrophages with neutrophils. The kidney is affected by severe, chronic active necrotizing granulomatous to pyogranulomatous nephritis

**Figure 3:**
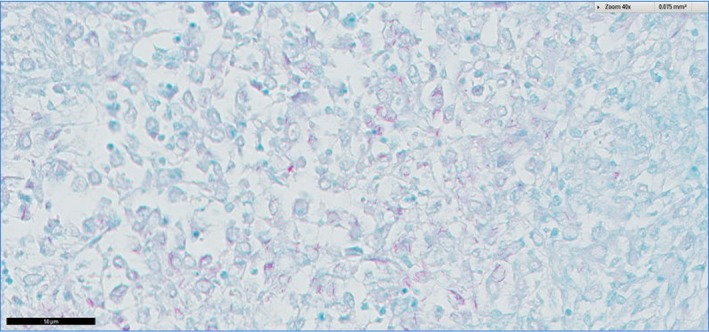
A section of kidney from Case 0 stained with Ziehl‐Neelsen stain which reveals moderate numbers of intracellular and extracellular acid‐fast organisms morphologically typical of *Mycobacteria* spp

Examination of haematoxylin and eosin stained sections showed extensive, multifocal to coalescing areas of severe, chronic‐active, necrotising, granulomatous to pyogranulomatous nephritis (Figure 2). Silver staining for the identification of spirochetes was negative, as was periodic acid‐Schiff staining for fungi (not shown), while a Ziehl‐Neelsen stain revealed moderate numbers of positive staining acid‐fast bacilli morphologically typical of mycobacteria within the cytoplasm of epithelioid macrophages, indicative of mycobacterial infection (Figure 3).

The Animal and Plant Health Agency (APHA) was notified of the findings and remaining fresh tissue was submitted for both routine mycobacterial culture (APHA, Weybridge) and for mycobacterial PCR (Leeds University Teaching Hospital Microbiology Department), both of which confirmed infection with *M. bovis* subsp. *bovis* which was genotyped to 10:a.

### 2.2 Testing regime

Once *M. bovis* infection had been confirmed a prospective test and cull policy was implemented at the kennels in order to contain the spread of infection within the pack and remove any potentially infected animals. This was combined with immediate voluntary movement restrictions implemented by the kennel and increased biosecurity measures, for example, increased kennel disinfection protocols, cessation of feeding fallen stock and immediate repairs to limit wildlife access to the hounds.

#### 2.2.1 Interferon‐gamma release assays

Interferon‐gamma (IFN‐γ) release assay tests were originally developed on the principle of quantitatively evaluating IFN‐γ production by peripherally circulating antigen‐specific effector memory T‐cells upon in vitro stimulation, in order to aid the diagnosis of *M. bovis* TB (bTB) in cattle, where it has a reported sensitivity of 81.8%–100% and specificity of 88%–99% (Bezos et al., 2014; Schiller et al., 2009; Vordermeier et al., 2006; Wood & Jones, 2001). They have subsequently been adapted to identify active and latent TB in human patients with at least equivalent sensitivity and increased specificity compared to the tuberculin skin test, as well as being practically easier to perform (Eisenhut, 2014; Kim et al., 2011; Thillai, Pollock, Pareek, & Lalvani, 2014; Zhou et al., 2011). An IGRA test has been validated for use in domestic cats with up to 100% sensitivity for the detection of MTBC infections (Rhodes, Gruffydd‐Jones, Gunn‐Moore, & Jahans, 2008a, 2008b; Rhodes et al., 2011). Whereas intra‐dermal testing has been shown to be of unreliable clinical utility in the dog, an IGRA has been used successfully for the detection of *M. tuberculosis* infections in dogs in a high risk setting (Parsons et al., 2012). The in vitro nature of the test protocol allowed us to test individual animal responses to multiple antigen combinations with technical replicates in this outbreak.

To interrogate the immune response of the hounds to mycobacteria, the antigens purified protein derivative (PPD) *M. avium* (PPDA)*,* PPD *M. bovis* (PPDB) and a cocktail of the RD‐1 specific immunodominant proteins ESAT6 and CFP‐10 were selected. PPDA from *M. avium* is frequently used in both IGRA and tuberculin skin testing to assess for exposure/sensitization to, or infection with, environmental mycobacteria species (Pai, Dheda, Cunningham, Scano, & O'Brien, 2007; Rhodes et al., 2008a; Wood & Jones, 2001). Infection with an MTBC mycobacteria is confirmed if the IFN‐γ response of any animal is greater to PPDB than to PPDA (Rhodes et al., 2008a; Wood & Jones, 2001). The presence of a concurrent response to the immunodominant antigens ESAT‐6/CFP‐10 indicates infection with an RD‐1 positive MTBC mycobacteria (i.e., excludes infection with *M. microti* or previous vaccination with *M. bovis*‐BCG) (Guinn et al., 2004; Teo et al., 2013).

The IGRA assays were conducted at Biobest Laboratories, Edinburgh in line with the assay protocol validated and previously published for use in the cat (Rhodes et al., 2008a, 2011). A 5 ml heparinized whole blood sample was taken from each hound and transported to the laboratory at ambient temperature within 18 hrs. Upon receipt, peripheral blood mononuclear cells (PBMC) were removed; blood was diluted 1:1 with Hanks Balanced Salt Solution (HBSS, Gibco, UK) and layered over Histopaque 1077 (Sigma, UK) before centrifugation at 800 *g* for 40 min at room temperature. PBMC were removed from the resulting interface, washed with HBSS and re‐suspended in complete culture media (RPMI 1640 containing 100 μg/ml L‐glutamine, 10% foetal bovine serum, 100 μg/ml penicillin, 100 U/ml streptomycin, 5 × 10^−5^ mol/L 2‐mercaptoethanol and non‐essential amino acids) to a density of 2 × 10^6^/ml. 100 μl of PBMC suspension was stimulated in duplicate with PPDA or PPDB (Lelystad, Prionics, Netherlands); both at a final concentration of 10 μg/ml, a peptide cocktail of ESAT‐6/CFP‐10 at a final concentration of 5 μg/ml (Lionex, Germany), a mitogen positive control of phorbol myristate acetate plus calcium ionophore (PMA/Ca, Sigma, UK, 50 ng/ml and 1 μg/ml respectively), and finally a complete culture medium only (i.e., negative) control.

Cells were incubated for 4 days at 37°C/5% CO_2_, after which the supernatants were removed, and duplicates pooled, for quantification of IFN‐γ by ELISA. Supernatants were either directly assayed or stored at −80°C until required. The IFN‐γ ELISA was conducted using a commercially available canine specific ELISA kit (DY781B, R&D Systems, Europe Ltd., UK) according to the manufacturer's instructions. Pooled supernatant from each cell culture condition was assayed in duplicate. Optical density (OD) values were measured at a wavelength of 450 nm and the replicate values for each condition were averaged and standard deviations calculated. Where replicates differed by more than 30% from each other, the test was considered invalid.

Prior to the instigation of testing, a prospective case definition was set to determine which hounds would be considered positive; it was decided that a statistically significant response to any of the three test antigens (PPDB, PPDA or ESAT6/CFP10) above the negative (culture medium control) condition was plausibly indicative of a biologically significant antigen specific T‐cell response. Furthermore, such a response would be consistent with not just exposure to mycobacteria but rather would be suggestive of significant challenge and probable infection. To achieve maximum diagnostic sensitivity, and due to the fact that very little is known about the infection dynamics, clinical progression or probability of mycobacterial shedding by dogs once they are infected with mycobacteria, it was assumed that any hound with a significant IFN‐γ response to at least one test antigen was infected and potentially infectious. Any such hound would therefore pose a risk to human and animal health as well as to the environment and so should be removed from the pack and euthanased.

As no cut off values or reference intervals exist for the dog, we defined the threshold for responsiveness to antigen stimulation as the mean OD value of the negative control replicates, plus two standard deviations ($\overset{¯}{x}+2SD$). Minimum antigen responses were defined as the mean OD value of the replicates, minus two standard deviations ($\overset{¯}{x}-2SD$). If a calculated minimum antigen response value was greater than that calculated for the negative control, then the response was considered positive. For a test to be considered interpretable, the PMA/Ca (positive control) response had to be positive by the same criteria when compared to the negative control.

In total 164 hounds were tested by IGRA, only 11 tests failed and required repetition; the most frequent reason for this (*n* = 6) was that there was insufficient response to the PMA/Ca positive control, followed by clotted blood samples which precluded the isolation of PBMC (*n* = 4) and only one test required repetition due to significant disagreement between replicate values. All IGRA test result data are shown in Supporting Information Table S1.

Of the 164 hounds, 85 (52.0%) were found to be positive by the above case definition. Within those found to be positive, 77 (90.1%) displayed a significant response bias to PPDB which was greater than the PPDA response (which was also significantly elevated above the negative condition in 37, 48.1%, of these hounds), a pattern considered indicative of MTBC infection.

Within the group of hounds displaying a typical MTBC infection response, 48 (62.3%) also responded to the peptide cocktail ESAT‐6/CFP‐10. Five of the test positive individuals (3.1%) responded to ESAT‐6/CFP‐10 and no other test antigens. These individuals were considered positive by the case definition and so were removed from the pack and euthanased. Similarly, only three individuals (1.8%) responded to PPDA and no other test antigens; whilst it was considered unlikely that these were infected with MTBC mycobacteria, they were removed from the pack, and euthanased, as a precaution.

#### 2.2.2 Serological assay

Concurrently to the IGRA testing, serological assessment of each hound was conducted. Blood was taken from each hound at the same time as the whole blood was collected for IGRA testing, 5mls of whole blood was placed into plain blood tubes and left upright at room temperature to allow clot formation. On arrival at the laboratory, blood was centrifuged at 800 *g* for 15 min at room temperature. Serum was removed and frozen at −20°C in 500 μl aliquots until needed.

The Dual Path Platform (DPP) VetTB test for Cervids (Chembio Diagnostic Systems Inc., USA) consists of two nitrocellulose strips inside a cassette that allows independent delivery of the test sample and antibody‐detecting reagent to the two test antigens (MPB83 and combined ESAT‐6/CFP‐10) and the test control (Jaroso et al., 2010). One cassette was used per hound. Tests were carried out at room temperature. Thirty microlitres of serum was dispensed into the sample well followed by two drops of sample buffer. After five minutes, a further four drops of sample buffer was added to the buffer‐only well. Cassettes were incubated for a further 20 min. This protocol is used by APHA for Eurasian badger (*Meles meles*) TB serology with preliminary estimates of test sensitivity of 55.3% (38%–71.4%) and specificity of 97.5% (86.8%–99.9%) (Greenwald et al., 2003). Test results were obtained by inserting each cassette into a hand‐held optical reader device (Chembio Diagnostic Systems Inc., USA), measuring reflectance in relative light units (RLU). A numerical RLU value for the control band (to show the test was valid) and the test band was provided by the reader.

Test results were obtained first qualitatively by visual inspection and to check the cassette quality control band and the presence/absence of antigen binding by serum antibody, and then quantitatively by inserting each cassette into an Optricon DPP Reader (Chembio Diagnostic Systems Inc., USA), to measure reflectance of antigen binding by antibody in relative light units (RLU).

A total of 163 hounds were tested; of these 11 (6.7%) generated positive test bands visible by eye; of these, nine had antibodies directed against antigen MPB83 and two against ESAT‐6/CFP‐10. No hounds were positive to both MPB83 and ESAT6/CFP10. Hounds which produced a visibly positive test were considered to be infected, removed from the pack and euthanized.

**Figure 4:**
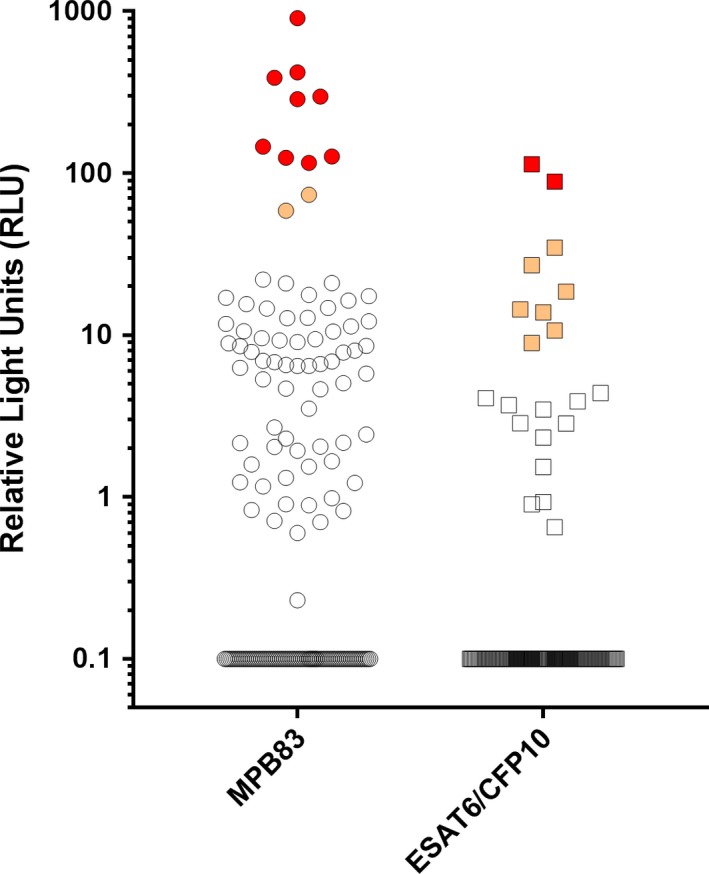
Dual Path Platform (DPP VetTB, Chembio, USA) serology test results (given as Relative Light Units [RLU]) for each of 163 hounds tested. Responses (RLU) to the two test antigens MPB83 (circles) and ESAT6/CFP10 (squares) are shown. Hounds producing a visible/qualitative test‐positive result are represented by red symbols, those giving an intermediate result are represented by orange symbols and test negative animals are shown as black open symbols. Negative samples scoring zero RLU were assigned as “0.1” to allow their appearance on this log‐scale graph

In addition, a further 10 hounds (6.1%) showed RLU values greater than that of the majority of the remaining population and were assigned as “intermediate positive” test results (Figure 4). While interpreting these results is challenging, these hounds were considered to be at increased risk of infection in comparison to the background population and so were also removed from the group and euthanased. All test result data for the Chembio DPP VetTB test are given in Supporting Information Table S2.

#### 2.2.3 Screening of at risk, in‐contact dogs

During the course of the outbreak, a number of dogs were identified out with the pack which had considerable potential for exposure to the hounds at the affected kennels. These dogs fell into three groups; (A) those dogs kept as pets by individuals who lived at or worked closely with the kennels and who spent a considerable amount of time with the hounds; (B) individual bitches from the affected kennel, that had been sent within the previous 18 months to other kennels for the purpose of breeding and remained at those kennels; and (C) a group of bitches that were sent from the affected kennel during the 18 months prior to the outbreak, to other kennels for purposes of breeding and were then returned, possibly bringing the infection with them.

After consultation with owners, 19 at‐risk pet dogs (group A) were subjected to IGRA testing according to the same protocol as the hounds, outlined above (Section 2.2.1). Of these dogs only two were found to produce significant responses to PPDB and with a PPDB > PPDA bias, neither of these were responsive to ESAT‐6/CFP‐10. Retrospective investigation revealed that one of these dogs was a hound which had been retired from the affected pack due to age‐related poor performance only a few weeks prior to Case 0 becoming clinically sick. The second was a terrier dog, used as a “cutting room scrap dog” that is, it was regularly close to the preparation of carcasses before they were fed to the hounds.

In total, 13 bitches were sent from the affected kennel to one of two other kennels for breeding purposes, (groups B and C); both of these kennels are in the High Risk Area (HRA) of endemic bTB of England. Seven of these bitches were still away at the other kennels at the time of testing (i.e., group B), where they were assessed by IGRA and DPP VetTB test assay (as outlined above; Sections 2.2.1 and 2.2.2) and found to be negative on both tests. Six bitches had been off site for breeding purposes in the 18 months preceding the outbreak and returned to the kennels before the first case occurred (i.e., group C). Only three of these animals were still alive at the time of the outbreak; one was found to be IGRA positive (PPDB > PPDA) but was DPP VetTB test‐negative and had no visible lesions at PME. The remaining two live hounds were test negative by both assays. The reason for, and timing of, euthanasia or death of the three untested hounds is not known as it was not recorded by kennel staff.

#### 2.2.4 *Post‐mortem* examination of test positive animals and subsequent mycobacterial culture

During the course of the outbreak, from December 2016 until July 2017, a total of eight hounds became clinically unwell (Table 1). All hounds displayed similar clinical signs to Case 0 including acute onset anorexia, lethargy, polyuria and polydipsia. Only one of these hounds was subjected to IGRA testing *ante mortem* and was found to be responsive to the antigen cocktail of ESAT‐6/CFP‐10, the remainder had to be euthanased for welfare reasons before samples could be obtained.

**Table 1:** Results of *post‐mortem* investigations compared to *ante mortem* test results

Initial screening tests				
	DPP positive	DPP “at risk”	DPP negative	Total
IGRA positive	4	5	76	86^a^
IGRA negative	7	5	66	78
Total	11	10	142	164

Repeat tests				
	Culture positive	Culture negative	Culture not performed	Total
IGRA positive	0	0	9	9^b^
IGRA negative	0	0	57	57
Total	0	9	57	66

*Post‐mortem* results (Hounds euthanased following initial screening)					
	Culture positive	Culture negative	Culture not performed	Total	Additional notes
Clinical signs, no *ante mortem* testing	7	0	0	7	Two hounds had histopathological renal abnormalities; chronic‐active granulomatous nephritis
Clinical signs; IGRA positive, DPP test not done	1	0	0	1	Significant IFN‐γ response was seen to ESAT‐6/CFP‐10 only. Lesions present at *post‐mortem* examination
IGRA positive, DPP positive	2	0	2	4	Visible lesions present at *post‐mortem* examination of culture‐positive hounds
IGRA positive, DPP “at risk”	2	0	3	5	
IGRA positive, DPP negative	1	17	58	76	
IGRA negative, DPP positive	1	2	4	7	
IGRA negative, DPP “at risk”	0	2	3	5	
IGRA negative, DPP negative	0	0	66	66	
Total	14	21	136	171	

*Notes*

DPP: Dual Path Platform; IGRA: interferon gamma release assay.

^a^One animal not DPP tested. ^b^PME of these nine hounds were unremarkable (see Results).

All IGRA and serology test positive but clinically healthy animals were euthanased and carcasses disposed of by incineration. Prior to incineration, all carcasses were subjected to a minimum of a gross PME; the exterior of the carcass was assessed, the abdominal and thoracic cavities were opened and all viscera visually inspected. Overall, 97 of the 164 hounds tested in the initial screening tests were euthanased. Of these 97, three (3.1%) were found to have grossly visible renal pathology (visible lesions, VL) as described for Case 0; lesioned tissues were removed for mycobacterial culture and histological examination. In all cases, acid‐fast bacilli were noted on histology and *M. bovis* was cultured from the fresh tissue (see below).

In addition, 24 (25% of the 97) non‐visible lesioned (NVL) hounds were randomly selected to have a full PME with samples taken for mycobacterial culture and histological examination. For each of these hounds the submandibular, pre‐scapular, axillary, bronchial and mesenteric lymph nodes were removed with one (left hand side for paired nodes) or one half (for unpaired nodes) formalin fixed and the other kept in sterile containers for mycobacterial culture. Given the frequency of renal lesions in previously confirmed cases, representative internal samples of both kidneys as well as urine obtained by cystocentesis were taken for mycobacterial culture.

*Post‐mortem* samples were assessed by mycobacterial culture either at APHA, Weybridge (clinically sick animals only) or the Roslin Institute, University of Edinburgh (clinically sick and clinically well animals). Samples from two of the eight clinically sick animals (25%) were tested in both laboratories to check for culture agreement and both laboratories were able to grow *M. bovis* from both samples.

All samples for mycobacterial culture were treated according to APHA's standard operating procedure by both laboratories. Briefly, inside a Containment Level 3 (CL3) laboratory up to 20 g of tissue was homogenized, decontaminated with 5% oxalic acid or 10% sodium hydroxide, and centrifuged, the pellet was re‐suspended in sterile phosphate‐buffered saline (PBS) and centrifuged again. Urine was centrifuged and removed from the sediment. The homogenate or urine sediment respectively was then re‐suspended in PBS and sown onto solid Middlebrook 7H11 OADC (Sigma, UK) and liquid Middlebrook 7H9 ADC (Sigma, UK) culture media.

Cultures were read at 6 weeks of incubation and again at 14 weeks in order to allow sufficient time required for any MTBC organism (i.e., either *M. bovis* or *M. microti*) to grow in culture if present in the tissue sample.

A total of 14 hounds (8.2%) were confirmed as infected with *M. bovis* by mycobacterial culture (Table 1). Of these; all eight hounds which showed signs of clinical disease were found to be culture positive at 6 weeks. Six hounds were sub‐clinically infected; three were identified as being visible lesion positive at PME, in each case *M. bovis* was isolated from at least one lymph node, at least one kidney and from urine samples. All cultures in these animals were positive by 6 weeks. Three hounds were NVL at PME but were culture positive, as with the VL hounds, in each case *M. bovis* was isolated from at least one lymph node, at least one kidney and from urine samples, however, all cultures were only found to be positive at 14 weeks.

Positive cultures were harvested and heat killed before genotyping; *M. bovis* was identified on the basis of colony morphology and genotyping by APHA. Genotyping was performed using spoligotyping (Kamerbeek et al., 1997) and VNTR typing (Exact Tandem Repeat loci A to F, Frothongham & Meeker‐O'Connell, 1998). Positive cultures grown at the Roslin Institute were harvested and digested with enzymatic lysis buffer containing 20 mg/ml lysozyme (Sigma, UK). Mycobacterial genomic DNA was obtained using a DNeasy Blood and Tissue kit (Qiagen, Germany) in accordance with the manufacturer's instructions. Harvested DNA was used as a template for PCR identification of the mycobacterial genes heat shock protein 65 (*hsp65*) and 16s rDNA. If these were positive further testing was conducted to look for the presence of the MTBC antigen 85A, and the RD‐1 specific gene ESAT‐6. A summary of all PME and culture results is shown in Table 1.

#### 2.2.5 Repeat interferon‐gamma release assays testing protocol

Once the initial screening testing had been completed, the 66 hounds that remained were kept in isolation for 60 days. At this time, because the test accuracy of the two testing methods is unknown, the IGRA test was repeated. Nine hounds were found to be IGRA positive, none were visibly lesioned at PME.

Following these results it was decided that the remaining 57 hounds were unlikely to be infected/infectious and so voluntary restrictions on the kennels were lifted. The hounds continue to be closely monitored for any change in appetite, body weight and body condition score. All of the remaining hounds remain well at the time of writing.

## 3 SCREENING OF AT RISK, IN‐CONTACT HUMANS

*Mycobacterium bovis* is a known zoonotic bacteria. Once infection was confirmed by laboratory culture from samples taken from the hounds, Health Protection England (HPE) was informed to assess the risk to human health. An in‐depth risk assessment was conducted, and contacts were stratified in to risk pool. A ‘stone in the pond’ approach was adopted for screening, with those at highest risk of exposure screened initially. This investigation and its findings are the subject of an additional study, where 11 people were screened (Phipps et al., submitted). One asymptomatic exposed person has tested positive for TB on initial screening by IGRA and has since been diagnosed with latent TB. Due to the nature of the contact between this individual and the infected hounds, it remains possible but unproven that the person was infected by contact with either the hounds and/or contaminated bovine material.

## 4 EPIDEMIOLOGICAL ASSESSMENT

### 4.1 Risk pathway identification

The APHA conducted an epidemiological investigation into the source of the outbreak to investigate possible transmission pathways. A qualitative risk assessment approach was used to address the likelihood of each of these routes of infection. The more probable contenders in order of likelihood were:

#### 4.1.1 Movement of infected hounds into the kennels (inhalation/ingestion/biting)

Thirteen female hounds moved to, and six returned from, other kennels for breeding purposes during the 18 months prior to clinical signs in Case 0. This included locations in the HRA that were also within the home‐range for genotype 10:a of *M. bovis*, where infected carcases would have been more likely to have been fed. Within the previous 3 years, but prior to the 18 months investigation in Section 2.2.3; three hounds, two male and one female, moved onto the premises from three other kennels in the HRA. The likelihood of these hounds becoming infected from fallen stock at these kennels or through other transmission pathways in the HRA was assessed as medium and therefore the most likely. This likelihood was somewhat mitigated by the fact that only one of the tested bitches was positive to the screening tests, and she had no visible lesions at PME. However, not all animals could be followed up because of missing records or mortalities, only ten of the 16 traced animals were alive and available for testing at the time of the outbreak.

#### 4.1.2 Feeding of *M. bovis*‐infected fallen stock (ingestion)

Details of the fallen stock collected from farms and fed to the hounds in the kennels over the 12‐month period prior to the first hound death were examined to provide an evaluation of the likely risk posed. A total of 24 carcases were sourced from an Approved Finishing Unit (AFU) for negative‐testing cattle from TB restricted farms. However, no tuberculous lesions had been confirmed at *post‐mortem* meat inspection in the abattoir in any cattle from the unit over the previous 3 years, reflecting the fact that most were sourced from low cattle TB incidence areas, that is, from across the Low Risk Area and/or low incidence parts of the Edge Area. Apart from the AFU, six carcases were collected from farms with a history of TB breakdowns caused by genotype 10:a of *M. bovis*—two dating back to breakdowns in 2014 and one in early 2016 due to the purchase of one infected cow. However, none of these incidents had ongoing infection within the herds in question and all had been subject to parallel IGRA and tuberculin skin testing, with negative results, which reduces the probability of residual infection.

The feeding of fallen stock from local cattle herds with undisclosed *M. bovis* 10:a infection remains a low likelihood considering the low TB incidence in the area, annual TB testing of herds, and the herd level test sensitivity with a cut‐off of one reactor. Only one farm was in a 4 year testing parish, but was assessed as low risk after considering its purchase history. No alpaca carcases or lungs had been fed. However, it is possible that kennel staff may not have recognized TB lesions in carcases if present. The likelihood of feeding infected material to the hounds at this kennel was consequently assessed as low, but with a medium level of uncertainty regarding prevalence of carcase infection and dose–response in dogs.

#### 4.1.3 Exposure to infected livestock or wildlife during exercising (inhalation/ingestion/biting) either directly or via *M. bovis*‐contaminated environment

About 85% of the geographical area to which the hounds were exposed had low cattle TB incidence and no indication of wildlife infection. The remaining 15% had some TB cattle breakdowns where APHA investigation indicated possible wildlife sources. The very infrequent reporting of *M. bovis* in farm dogs suggests that farm‐based transmission pathways are not very effective (Wilkins et al., 2008). Kennel staff reported that direct contact between wildlife and working hounds during exercise was unlikely. However, the worst case scenario of an encounter with an infectious badger, or infected carcase could have been missed. The likelihood of infection whilst exercising was considered very low, but with high uncertainty associated with wildlife prevalence and general lack of quantification of the transmission pathways from the environment.

#### 4.1.4 Exposure to infected local wildlife at the kennels (inhalation/ingestion/biting)

The kennels are located in an area of the country where cattle TB incidence is low and there is no evidence of wildlife infection. Access to the kennels by larger wildlife such as badgers was very unlikely. Likelihood was considered very low with low uncertainty.

Once in the kennels, infection appears to have spread horizontally between the hounds, suggesting that in certain conditions such as intensive housing of large packs, relatively high rates of transmission can occur within dog populations. It is known that badgers can excrete up to 100,000 *M. bovis* colony forming units (CFU) per ml of urine where kidneys are infected (Corner, O'Meara, Costello, Lesellier, & Gormley, 2012). Dogs with similar pathology may have similarly high excretory rates. Inhalation of infected urine aerosols or ingestion of such high doses are valid and likely transmission pathways in this scenario to account for dog to dog spread. However, the fact that no gross tuberculous pathology was found in the respiratory and digestive systems of the clinical cases or test positive dogs adds a level of uncertainty as to what the exact transmission route was.

### 4.2 Statutory changes resulting from the assessment

This outbreak prompted a policy review of the feeding of raw flesh from fallen stock to hounds, permitted under Article 18 of Commission Regulation (EC) No. 1069/2009. A condition of this regulation is that fallen stock fed are not killed or have not died as a result of the presence or suspected presence of a disease communicable to humans or animals. An interpretation of this is that TB reactors, normally removed by APHA, and inconclusive reactors, should not be fed. The Department for Environment, Food and Rural Affairs (DEFRA) commissioned a risk assessment from APHA to consider the likelihood of *M. bovis* infection occurring in hounds at Animal By‐Product (ABP) registered kennels through the routine feeding of fallen stock sourced from farms in the HRA of England.

The overall likelihood of at least one foxhound becoming infected with *M. bovis* within the next 5 years, if current practices remained unchanged, was considered to be medium. This assessment was subject to a high level of uncertainty associated with the true prevalence of *M. bovis* infection in fallen stock carcases in the HRA and the dose–response following ingestion of infected material in dogs. However, the fact that this is the first reported case in a kennel in the UK, may suggest that the actual risk is lower. On the one hand this conclusion is caveated by the possibility of under‐reporting through lack of systematic surveillance for TB in dogs. Alternatively, the conclusion is supported by a survey of culled hounds from ten Irish hunting kennels, over a 3‐year period (2003–2005), which identified *M. bovis* infection in one of 52 foxhounds submitted for PME as part of the survey (Jahns, Callanan, McElroy, Sammin, & Bassett, 2011).

As a proportionate risk mitigation strategy, DEFRA has introduced tighter restrictions on the collection and feeding of fallen stock to hounds in registered kennels. Since 10 October 2017 the feeding of offal from livestock species to dogs from recognized kennels or packs of hounds has been banned in England (Anonymous, 2017). Hunt kennel operators must also carry out additional examinations for lesions of TB in fallen stock originating from “high risk” premises (defined as farms under movement restrictions due to a bovine TB breakdown, or which have been released from such restrictions in the previous 12 months). In addition, APHA has developed training materials and guidance on the identification of TB in carcases of cattle and other livestock, for collectors and kennels feeding fallen stock to hounds. This should raise awareness of TB and assist recognition of tuberculous lesions in fallen stock for notification of suspicion of TB in carcases to APHA, as legally required under The Tuberculosis (England) Order 2014. The hunt kennel industry is also strengthening the voluntary code of practice and guidance for its members, to recommend PME of all hounds that die of unexplained or suspect causes.

## 5 DISCUSSION

To the authors’ knowledge this outbreak represents the only documented occurrence of TB in a canine species with evidence of onward dog‐to‐dog transmission within the affected group. Previous reports of TB in dogs have been of single sporadic cases or small case series of unrelated infections. The reason for, and source of, this outbreak remains unproven; however, given the known ability of *M. bovis* to spread readily between social animals, it is not surprising that a large group of kennelled hounds presented a pool of competent, susceptible hosts once the pathogen was introduced. Also remarkable in this outbreak was its fulminant nature; from the occurrence of the index case until the resolution of the outbreak (7 months), nearly one in ten animals on the premises became clinically sick. TB is usually considered a chronic disease with only a small percentage of infected individuals becoming clinically unwell (Eisenhut, 2014). Therefore, this rapid progression to disease in a relatively high proportion of animals can be considered unusual, although not unheard of, as sporadic fulminant human cases of renal TB leading to death by acute renal impairment have been reported (Adzic‐Vukicevic et al., 2017; Dissanayake, 2004; Isao et al., 2006; Pathan et al., 2001; Punia & Kumar, 2008; da Silva Junior, Brito, Rabelo, & Saboia, 2016).

The reasons for the fulminant nature of this outbreak are likely to be multifactorial; a combination of pathogen virulence, host genetics and phenotype, as well as environmental conditions. There is a possibility that the pathogen became host‐adapted. However, all animals from which viable organism was isolated were found to be infected with genotype 10:a. It may therefore also be the case that this strain of *M. bovis* in particular poses some genetic virulence with respect to the dog, or that it became tolerant to a new host and so spread amongst the hounds once introduced.

From a host perspective of transmission dynamics, the kennel stocking density was higher than that recommended by the Masters of Foxhounds Association (MFHA). This would have created an ideal opportunity for spread of disease once the index case became clinically sick and infectious. Similarly, the physiological stress of overcrowding may have made individuals more susceptible to infection. A number of the kennels were in suboptimal states of repair. This meant that once the kennels became contaminated, it was almost impossible to appropriately disinfect them. They have since been destroyed completely and the kennel has moved to a new, clean site. Foxhounds *per se* are known to be at risk of mycobacterial infections with outbreaks of canine leproid granulomas reported in New Zealand and Australia (Smits et al., 2012). These outbreaks all affected closely related individuals and so the high level of genetic relatedness between individuals within this pack could imply that if a genetic susceptibility, such as a defect in cellular immune response, did exist, then it would likely have high penetrance.

The majority of the clinical signs in sick hounds were non‐specific and compatible with those seen due to *M. bovis* infections in other species; lethargy, anorexia, weight loss and lymphadenopathy (Isaac, Whitehead, Adams, Barton, & Coloe, 1983; Murray et al., 2015; O'Halloran & Gunn‐Moore, 2017). Unusually for companion animals, every clinically ill animal, those with lesions at PME as well as NVL hounds that were found to be culture positive, had renal infections. The kidney is not generally considered a so‐called “target organ” for mycobacterial infections in companion animals, however, it is the second most common site of extra‐pulmonary TB in humans, after lymph nodes (Adzic‐Vukicevic et al., 2017; Murray et al., 2015; O'Halloran & Gunn‐Moore, 2017; Pesciaroli et al., 2014; da Silva Junior et al., 2016). Renal tuberculous infections are often considered to be insidious and present with mild or even subclinical disease, a significant proportion of cases are incidental findings at PME (Guarino, Martínez‐Roig, Maiques‐Llacer, González‐Rivero, & Anguerri‐Feu, 2009; Isao et al., 2006; Punia & Kumar, 2008). In human cases of infection where irreversible kidney damage occurs, there are commonly co‐morbidities. Amyloidosis is one such co‐morbidity, particularly recognized across India, which predisposes to glomerulonephritis and potentially fatal nephrotic syndrome (Chong et al., 2017; Sanz‐Martín, Samillán‐Sosa Kdel, De Miguel, & Martínez‐Miguel, 2016). This may also be the case for dogs; Foxhounds have previously been reported to be affected by renal amyloidosis (Mason & Day, 1996), and this kennel has had previously confirmed clinical cases, and one of the *M. bovis* infected hounds was found to be positive for renal amyloidosis (data not shown).

Other conditions which adversely affect the outcome of renal TB are the deposition of anti‐mycobacterial antibodies within the glomeruli and/or hypercalcaemia which is a common complication of TB and is directly nephrotoxic (Bellendir, Kochanova, & Nakonechny 1978; Chan et al., 1994; Ko et al., 2004; Zhao, Sun, Xu, & Sun, 2017). It is possible that either of these conditions could have had an impact on the number of dogs affected and the severity of disease seen in this outbreak.

The relatively high level of renal immune‐privilege may have allowed mycobacteria to replicate there whilst avoiding immune surveillance and clearance (Kurts et al., 2001). All of the hounds with renal lesions also had positive urine cultures (where these were available), which suggests that this could be a major source of infection for onward dog‐to‐dog transmission. However, if contaminated urine was the main source of infectious material, for example, via aerosolization, then lung pathology would be expected, but there were no pulmonary lesions in any of the dogs subjected to PME. In addition, the urine cultures were only found to be positive after 14 weeks of culture suggesting that the initial number of organisms present was low. Based on the distribution of lesions it would appear that the mycobacteria arrived in the kidney by haematogenous spread. If so this indicates a distant source of infection which has not been identified.

Infected urine was identified as a key risk factor for potential human exposure during the risk assessment of kennel staff, predominately due to the use of power washers to clean excreta from the hard standing, which posed a significant risk of aerosolization (Phipps et al., submitted). The outcome of TB screening for at‐risk humans at the kennels was that a single individual was diagnosed with latent TB (Phipps et al., submitted). If urine from infected dogs was heavily contaminated with *M. bovis* it would have been likely that a higher proportion of individuals at risk would have been test positive. Based on these observational data, it is possible to conclude that *M. bovis* infected dogs are likely to be infectious to each other, and their urine may contaminate their local environment, but that this may not be the only or even the major route of transmission, and that the risk to human health is low.

No *ante mortem* tests have been validated for the diagnosis of *M. bovis* infection in dogs; we therefore combined two established testing methods for other species to try and maximize our diagnostic sensitivity and bring this outbreak under control. The tests used here target the disparate arms of the immune response, with the IGRA evaluating the cell mediated Th1 response whilst the serological assay used to detect a humoral Th2 response. The percentage of tested animals deemed to be positive by each test was markedly different with 52% of dogs classed as infected by IGRA at initial screening but only 12.9% of dogs classed as infected or “at risk” by serology. This difference is likely due to the dominance of cell mediated immunity in an animals’ response to mycobacterial infection (Khan, Hunter, & Jagannath, 2016) but further investigation of the canine immune response to *M. bovis* and mycobacterial antigens is needed before this can be shown conclusively, this work is currently ongoing and will be published separately.

Whilst uncertainty exists with regards to the source of infection, it seems likely that contaminated fallen stock carcasses were involved. All culture positive animals had the same genotype isolated, which has not been frequently detected in the area local to the kennels. This suggests a single introduction with a large dose of infectious material was likely and that infection then spread between cohabiting hounds (Reynolds, 2006). One kennel worker was also diagnosed with latent TB, potentially due to exposure to infected hounds and/or their contaminated feed, though this remains unproven. Though the risk posed by *M. bovis* infected hounds to other hounds, cattle herds, local wildlife, their environment and their human keepers is low, this outbreak demonstrates that the risk is plausible and real. Resultantly, to try to prevent such an outbreak from occurring again, the conditions for the collection and use of animal by‐products for feeding to dogs in recognized kennels or packs of hounds have now been amended to ban the feeding of offal from fallen stock. Similarly, health surveillance requirements for hounds have been increased by the MFHA. To be successful, these policy changes will need to be aided, however, by the future evaluation of diagnostic test accuracy and test development for canine TB.

## CONFLICT OF INTEREST

The authors declare no conflicts of interest.
